# A descriptive study of UK cancer genetics services: an emerging clinical response to the new genetics

**DOI:** 10.1054/bjoc.2001.1893

**Published:** 2001-07

**Authors:** D Wonderling, P Hopwood, A Cull, F Douglas, M Watson, J Burn, K McPherson

**Affiliations:** Cancer and Public Health Unit, London School of Hygiene and Tropical Medicine, Keppel Street, London, WC1E 7HT; The Cancer Research Campaign Psychological Medicine Group, Christie Hospital NHS Trust, Wilmslow Road, Withington, Manchester, M20 4BX; Imperial Cancer Research Fund, Psychology Group, Western General Hospital, Outpatient Building, Edinburgh, EH4 2XU; Department of Medical Genetics, Regional Genetics Service, 19/29 Claremont Place, Newcastle-upon-Tyne, NE2 4AA; Psychological Medicine, Royal Marsden Hospital and Institute of Cancer Research, Downs Road, Sutton, Surrey, SM2 5PT

**Keywords:** genetic counselling, cancer genetics services, health service organization, Calman-Hine

## Abstract

The objective was to describe NHS cancer genetic counselling services and compare UK regions. The study design was a cross-sectional study over 4 weeks and attendee survey. The setting was 22 of the 24 regional cancer genetics services in the UK NHS. Participants were individuals aged over 18 attending clinics at these services. Outcome measures were staff levels, referral rates, consultation rates, follow-up plans, waiting time. There were only 11 dedicated cancer geneticists across the 22 centres. Referrals were mainly concerned with breast (63%), bowel (18%) and ovarian (12%) cancers. Only 7% of referrals were for men and 3% were for individuals from ethnic minorities. Referral rates varied from 76 to 410 per million per annum across the regions. Median waiting time for an initial appointment was 19 weeks, ranging across regions from 4 to 53 weeks. Individuals at population-level genetic risk accounted for 27% of consultations (range 0%, 58%). Shortfalls in cancer genetics staff and in the provision of genetic testing and cancer surveillance have resulted in large regional variations in access to care. Initiatives to disseminate referral and management guidelines to cancer units and primary care should be adequately resourced so that clinical genetics teams can focus on the genetic testing and management of high-risk families. © 2001 Cancer Research Campaign http://www.bjcancer.com


*British Journal of Cancer* (2001) **85**(2), 166–170
© 2001 Cancer Research Campaign
doi: 10.1054/ bjoc.2001.1893, available online at http://www.idealibrary.com on IDEAL®

http://www.bjcancer.com

### A descriptive study of UK cancer genetics services: an emerging clinical response to the new genetics

**D Wonderling[1], P Hopwood[2], A Cull[3], F Douglas[4], M Watson[5], J Burn[4] and K McPherson[1]**

[1]Cancer and Public Health Unit, London School of Hygiene and Tropical Medicine, Keppel Street, London, WC1E 7HT; [2]The Cancer Research Campaign Psychological Medicine Group, Christie Hospital NHS Trust, Wilmslow Road, Withington, Manchester, M20 4BX; [3]Imperial Cancer Research Fund, Psychology Group, Western General Hospital, Outpatient Building, Edinburgh, EH4 2XU; [4]Department of Medical Genetics, Regional Genetics Service, 19/29 Claremont Place, Newcastle-upon-Tyne, NE2 4AA; [5]Psychological Medicine, Royal Marsden Hospital and Institute of Cancer Research, Downs Road, Sutton, Surrey, SM2 5PT

**Summary** The objective was to describe NHS cancer genetic counselling services and compare UK regions. The study design was a cross-sectional study over 4 weeks and attendee survey. The setting was 22 of the 24 regional cancer genetics services in the UK NHS. Participants were individuals aged over 18 attending clinics at these services. Outcome measures were staff levels, referral rates, consultation rates, follow-up plans, waiting time. There were only 11 dedicated cancer geneticists across the 22 centres. Referrals were mainly concerned with breast (63%), bowel (18%) and ovarian (12%) cancers. Only 7% of referrals were for men and 3% were for individuals from ethnic minorities. Referral rates varied from 76 to 410 per million per annum across the regions. Median waiting time for an initial appointment was 19 weeks, ranging across regions from 4 to 53 weeks. Individuals at population-level genetic risk accounted for 27% of consultations (range 0%, 58%). Shortfalls in cancer genetics staff and in the provision of genetic testing and cancer surveillance have resulted in large regional variations in access to care. Initiatives to disseminate referral and management guidelines to cancer units and primary care should be adequately resourced so that clinical genetics teams can focus on the genetic testing and management of high-risk families. © 2001 Cancer Research Campaign http://www.bjcancer.com

**Keywords**: genetic counselling; cancer genetics services; health service organization; Calman-Hine

There has been intense media publicity for cancer genetic discoveries, especially those relating to breast cancer (*BRCA1* and *BRCA2*), resulting in an increased public demand for information, reassurance and cancer screening.

In the UK, most regionally based clinical genetics centres now provide genetics clinics that are specifically for familial cancers. These services often involve collaboration between clinical geneticists, oncologists and other specialists. They have developed in a variety of ways according to local expertise and funding. In addition, a number of other family history clinics have been set up by other clinicians, such as breast surgeons, with variable levels of training in clinical genetics. A cancer genetics service will offer or recommend some of the following: risk estimation (based on molecular genetic analysis or more often on family history); genetic risk counselling; clinical examination; screening/surveillance for early tumour detection (mammography, endoscopy, etc); information on preventative strategies (surgery, diet, etc); family planning advice; and referral for psychological assessment/support (Murday, 1994; Ponder, 1994; Eeles and Murday, 1996).

In 1996, the Department of Health set up a working group on cancer genetics services chaired by Professor Peter Harper. The Harper report (Working Group for the Chief Medical Officer, 1998) recommended organization of cancer genetic services for England and Wales in a 3-tier structure integrated with the developing 3-tier cancer service that followed the Calman-Hine Report (Expert Advisory Group on Cancer, 1995). First, the role of primary care would be to refer on individuals newly identified as being at potentially high genetic risk of cancer, to follow-up existing high-risk individuals and to reassure individuals at population-level risk. Second, cancer units (based at district general hospitals) staffed by oncologists and surgeons with an interest in genetics, would provide risk assessment and, if appropriate, screening for individuals at moderately increased risk. Third, specialist genetics services would be integrated into the specialist cancer centre, serving a population of 1–2 million. This specialist service would be led by a consultant with training in both oncology and genetics, supported by 2 clinical nurse specialists (or one nurse and one clinical assistant). The specialist service would deal with high-risk individuals and would provide referral and management guidelines for the primary care and cancer unit teams within its region. The Harper report forms the basis of the NHS Cancer Plan's framework for cancer genetics services (NHS Executive, 2000).

An alternative service organization model has been proposed for Scotland (Priority Areas Cancer Team, 1998). Unlike the Calman-Hine scheme, there is no second tier. Instead staff from the regional genetics service would support staff in surveillance units and in primary care facilities by carrying out genetic counselling in outreach clinics. The service in Northern Ireland is modelled on the Calman-Hine system (Morrison and Nevin, 1999), but is developing centralized referrals mechanism, along the lines of the Scottish services.

Following the identification of cancer-predisposing genes, the national picture of cancer genetics service activity has been unclear. Reported in this paper are the results of a cross-sectional study conducted in 1998 showing the pattern of cancer genetics services across the NHS.

*Received 18 August 2000*
*Revised 29 March 2001*
*Accepted 5 April 2001*

*Correspondence to:* P Hopwood

### METHODS

All regional genetics services in the UK were invited to participate. Procedures were explained at a training workshop to help ensure good compliance. Ethical approval was obtained through the Multicentre Research Ethics Committee.

During a pre-specified 4-week period cancer genetic activity in all participating centres was logged. Designated staff-members at each centre completed report forms for every referral received, every individual attending, every telephone consultation and every cancellation/did-not-attend. Only activity relating to subjects under the age of 18 was excluded from the study. Information was requested about types of cancer/syndrome, source of and reason for referral, content of consulation and risk management plan for the individual. In addition, the lead consultant at each study centre was asked to supply general information about their service, such as catchment population size, frequency of clinics and details of staffing. Clinic attendees gave written informed consent and were asked to complete a questionnaire to provide mainly sociodemographic information.

We tested to see if the variability observed between regions could have occurred by chance. The Kruskal–Wallis test was used for waiting time and $\chi^2$ test for all other comparisons.

### RESULTS

22 of 24 regional genetics services agreed to participate. The catchment area of the participating centres covered the whole of Scotland, Wales and Northern Ireland and most of England, varying by region from 0.5 to 5.2 million population. These regional genetics services see individuals with a family history of cancer in a total of 141 hospitals. 53 of these hospitals held clinics specifically for familial cancer. 58% of new-attendee consultations were held in these designated cancer genetics clinics, 25% were in general genetics clinics and the remaining 17% were in other hospital clinics (e.g. breast lump diagnostic clinics). In the absence of facilities specifically for cancer families, there were 2 regions where all attendees were seen in general genetics clinics. In the other 20 centres the number of designated cancer genetics clinics provided per month ranged from 3 to 16 with a mean of 7.5.

The regions also varied according to whether they provided cancer screening (e.g. mammography) directly (3/22) or else referred on or left the clinical management decision with the general practitioner. There was also variability in the use of family history questionnaires as part of the referral process. 7 centres sent questionnaires to more than two thirds of referred individuals before deciding whether to offer an appointment. One would expect these centres to be seeing proportionately fewer people than those centres with less restrictive appointment criteria.

12 of the 22 lead clinicians from the centres were aware of other cancer family history clinics within the region but outside of their jurisdiction. They specified 13 such clinics organized by breast screening units, 14 by breast surgeons and 2 by bowel surgeons, although these may represent the tip of the iceberg. In Scotland the clinicians reported that almost all family history clinics were organized by the regional genetics service.

### Staffing of centres

In total there were 17 whole-time equivalent (WTE) consultants for a total catchment population of 53 million, consisting of 11 ‘dedicated’ cancer genetics consultants (those spending more than 50% of their working time on cancer genetics) and another 46 consultants (mainly clinical geneticists) allocating less time. This amounts to 0.3 WTE consultants, or 0.2 ‘dedicated’ consultants, per million of population. There were another 5 ‘dedicated’ cancer genetics clinicians below consultant grade and 33 genetic counsellors (clinical nurse specialists, genetic associates, etc.). Therefore only half of the centres had a ‘dedicated’ cancer genetics consultant and 6 had no such dedicated staff at all, including the one with the largest catchment population.

### Activity levels and case-mix

Table 1 shows the activity levels of the 22 regional centres combined. Referrals came equally from GPs (49%) and hospital clinicians (47%) with a few self-referrals. Table 2 shows the breakdown of referrals by gender and cancer site. The mean age of referrals was 41 years (SD = 11.5). Only 3% of attendees reported an ethnicity other than ‘white’ and almost half of these were ‘Jewish’ (a group known to be at greater genetic risk of breast cancer (Warner et al, 1999)).

Of the new-attendee consultations, 17% of attendees had previously been diagnosed with cancer (i.e. were ‘affected’). Clinicians were asked to assign new attendees to one of 3 risk level categories – in some cases this was imprecise because the clinician was waiting for additional information before making the final clinical management decision. One quarter of clinic attendees (26%) were categorized as ‘population level cancer risk or marginally above’. 49% were categorized as ‘risk level sufficient for screening’ and the remaining 25% was placed in the highest risk category.

**Table 1** Activity in 22 NHS cancer genetics services

| | Number in 4 weeks | Estimated number per year* | Estimated number per year per million population† |
|---|---|---|---|
| New-attendee consultations | 694 | 8744 | 164 |
| Followup consultations | 357 | 4498 | 84 |
| Home visits | 74 | 932 | 17 |
| Telephone consultations | 206 | 2596 | 49 |
| *All contacts* | *1331* | *16771* | *315* |
| Cancellations | 98 | 1235 | 23 |
| Failed-to-attend | 146 | 1840 | 35 |
| Referrals | 872 | 10987 | 206 |

*Assuming 252 clinic days per year (260 weekdays minus 8 public holidays). †Population = 53.3 million (90% of the UK).

**Table 2** Referrals, by cancer site and sex

| Referrals | Male | Female | All | |
|---|---|---|---|---|
| Breast* | 7 (1%) | 526 (99%) | 533 | 61% |
| Bowel† | 43 (32%) | 93 (68%) | 136 | 16% |
| Breast and bowel | 1 (5%) | 18 (95%) | 19 | 2% |
| Ovary‡ | 0 | 106 (100%) | 106 | 12% |
| Other specified§ | 12 (24%) | 37 (76%) | 49 | 6% |
| Not specified | 1 (4%) | 25 (96%) | 26 | 3% |
| All | 64 (7%) | 805 (93%) | 869 | 100% |

*Including BRCA1, BRCA2, Li-Fraumeni's syndromes. Individual may also include family history of other cancers but not bowel. †Including FAP, HNPCC, Lynch2 syndromes. Individual may also have family history of other cancers but not breast. ‡Individual may also include family history of other cancers but not bowel or breast. §Various cancer sites specified.

### Risk management

74% of attendees at marginal risk were to be discharged without follow-up compared with only 12% and 5% in the middle and upper risk categories respectively. 25% of the middle risk group and 30% of the high-risk group would be referred on to another specialist. Many of these onward referrals, 50%, were for screening (mammography, clinical breast examination, endoscopy, etc.), a few were clearly for prophylactic surgery (mastectomy, colectomy, etc.) but for the rest it was not clear whether the referral was for screening, surgery or some other purpose. Cancer screening would be recommended for 97% of moderately increased and high-risk individuals (continued for 47% and initiated for 50%). For those at population risk 13% of familial breast cancer cases and 44% of familial bowel cancer were already being screened and for these the recommendation would be to discontinue screening. Prophylactic surgery would be discussed with 30% of individuals estimated to be at high-risk of breast cancer and 19% of those at high risk of bowel cancer. Clinicians indicated that they would consider molecular genetic testing for 63% of high-risk individuals and 24% of those at moderate risk. The number of tests actually carried out would be much smaller because of supply constraints and because many individuals decide not to go ahead with testing after counselling (Peshkin and Lerman, 1999).

### Regional variations

Table 3 shows variations in activity between the regional cancer genetics services. Referral rates per million population varied significantly across the regions ($P < 0.001$) with a 5-fold difference at the extreme. Likewise, there was an 8-fold difference in the number of consultations with new attendees per million population across the region ($P < 0.001$). Referral rates were 53% higher in those centres providing mammography directly ($P < 0.001$) and 75% higher in those centres with a 'dedicated' cancer genetics consultant ($P < 0.001$). The 2 centres without familial cancer clinics had a 24% lower referral rate ($P < 0.043$). Referral rates were 40% higher in those centres with the most extensive use of pre-appointment questionnaires ($P < 0.001$), but they saw only 73% of referrals compared with 90% in the other centres. The proportion of a centre's referrals coming from primary care varied from 27% to 70% ($P < 0.001$).

There was a 13-fold difference in median waiting time across centres (range: 4–53 weeks; $P < 0.001$) (Table 3), but no significant difference in waiting time between high-risk and population-risk

**Table 3** Regional variations in cancer genetic activity

| Centre number† | Estimated New attendees seen p.a. per m | Estimated Referrals p.a. per m | Estimated Follow-ups p.a. per m | Median waiting time (weeks) | Proportion of attendees at low risk |
|---|---|---|---|---|---|
| 19 | 378 | 353 | 403 | 26 | 58.3% |
| 11 | 473 | 410 | 236 | 18 | 57.9% |
| 22 | 113 | 76 | 0 | 7 | 37.5% |
| 18 | 504 | 336 | 557 | 17 | 8.8% |
| 16 | 142 | 95 | 32 | 44 | 28.6% |
| 20 | 63 | 213 | 0 | 50 | 0.0% |
| 3 | 196 | 105 | 14 | 14 | 33.3% |
| 21 | 107 | 76 | 32 | 8 | 37.5% |
| 6 | 200 | 298 | 80 | 29 | 23.1% |
| 14 | 206 | 286 | 0 | 18 | 10.7% |
| 8 | 168 | 131 | 16 | 53 | 22.2% |
| 13 | 239 | 302 | 18 | 18 | 34.1% |
| 4 | 135 | 204 | 13 | 19 | 33.3% |
| 10 | 165 | 130 | 52 | 4 | 23.1% |
| 17 | 264 | 268 | 309 | 22 | 31.6% |
| 5 | 104 | 166 | 29 | 10 | 29.2% |
| 1 | 228 | 252 | 11 | 19 | 22.9% |
| 12 | 85 | 129 | 24 | 21 | 26.3% |
| 7 & 15* | 298 | 365 | 33 | 20 | 23.8% |
| 9 | 227 | 300 | 269 | 28 | 20.7% |
| 2 | 145 | 208 | 41 | 15 | 25.0% |
| **All** | **183** | **212** | **82** | **19** | **26.6%** |
| Scotland only | 289 | 278 | 203 | 18 | 32.6% |

*Centres 7 & 15 do not have distinguishable catchment populations. †Centres are arranged in ascending order of catchment size.

© 2001 Cancer Research Campaign subjects. Neither the use of questionnaire-based referral systems nor the availability of specialist cancer genetics staff appeared to affect waiting time but median waiting time was 5 weeks longer (22 weeks) for the 3 centres that provided screening directly ($P < 0.001$).

The proportions of attendees at different risk levels varied significantly ($P < 0.001$), with the proportion at population level risk ranging from 0 (two centres) to 58%. As GPs referred more individuals at population-risk than did hospital clinicians (29% cf 18%, $P < 0.001$), the variations in casemix between centres are partly explained by the variations in the source of referrals. Centres with questionnaire-based referral systems seemed to be seeing the same proportion of population-risk individuals as other centres. The same was true for those centres with a dedicated cancer genetics consultant.

The proportion of those at elevated risk who were offered genetic testing varied from 0 (2 Scottish centres) to 80% across the regions ($P < 0.001$) but the advice on screening (whether to initiate, avoid, continue or discontinue) did not differ significantly. Testing and screening were no more likely in centres with a dedicated cancer genetics consultant or in those that provide screening directly or in those without designated cancer genetics clinics. ‘Followup’ rates depended on screening strategy. The 3 centres providing mammography directly accounted for two thirds of all contacts with returning attendees.

Figures for the 4 Scottish centres combined are presented separately as the Scottish service model implies less restrictive referral criteria. There were almost twice as many referrals and consultations per million population in Scotland than in the rest of the UK. There were substantially more population-risk (33% c.f. 26%) and medium-risk individuals (54% c.f. 49%). The Scottish centres reported that genetic testing was only available for research purposes; consequently they reported that genetic testing would only be discussed with 10% of individuals at medium risk or above compared with 73% in the rest of the UK.

### DISCUSSION

### Access to cancer genetics services

All of the UK regional clinical genetics services deal with individuals with a family history of cancer and only 2 of the 22 regions surveyed did not have designated cancer genetics clinics. Many of these services have developed intensively over recent years and service provision compares favourably with that reported for the USA 4 years earlier (Thompson et al, 1995).

There is considerable variation in the resources and output of these services. The numbers of individuals referred were proportionately greater in those regions with a ‘dedicated’ cancer geneticist, direct provision of screening or questionnaire-based referral systems. Across the UK there was an 8-fold difference in the number of individuals seen as a proportion of catchment population and a 13-fold difference in waiting time, suggesting regional inequity of access. For high-risk families, those with a pattern of cancer in their family indicating Mendelian inheritance, access to the clinical genetics service is a greater priority since molecular testing in this group is more likely to find a genetic mutation. This would allow individuals at very high risk to be distinguished from relatives at much lower risk enabling more specific management. There were too few individuals at high-risk in this sample to effectively analyse regional equity of access for this subgroup. However, when this group is combined with those at moderate risk there is strong evidence of regional inequality of access to a specialist cancer genetics consultant and to genetic testing. There was no evidence of variation in screening recommendations, however this is somewhat tautological given that the moderate risk category was defined in relation to its need for screening. More important in terms of access is the supply of screening for genetically high-risk individuals, which in all but three regions is down to the individual policies of the local screening units and surgical departments.

A striking feature of the study was that referrals to and consultations at cancer genetics services overwhelmingly involve women and in particular those with a family history of breast cancer. This is partly explained by the prevalence of familial breast cancer in the population but must also be due to the publicity given to the breast cancer gene and to the interest in familial cancer shown by breast clinicians and geneticists over the last 2 decades. However, even within cancers that are not gender-specific, such as colon cancer, the number of women attending far outweighs the men. It is often thought that men generally under-use health services relative to women but the evidence is unclear (Moynihan, 1998). Women are sometimes described as the ‘guardians of family health’. The extent to which relevant genetic risk information will be passed on to other family members who may be at risk is not known. We observed, however, that only 35% of women (and 58% of men) reported that one of the reasons for attending was to find out the risk of other family members. Ethnic minorities also seem to be under-represented. The breakdown by gender and cancer site is remarkably similar to that of France (Sobol et al, 1999) and comparable with other European services (Hodgson et al, 1999). However, there was a much larger proportion of affecteds in France, as a result of cancer genetics being carried out by cancer centres rather than genetics centres. Cancer genetic activity in France (2500 new cases each year) was barely a quarter of our estimate for the UK (Table 1).

### Achievement of service guidelines

There are few doctors who specialize in cancer genetics. According to the Harper Report recommendations, there should be at least 27 dedicated cancer genetics consultants for the population covered in this study, compared with the 11 observed. This situation is unlikely to change in the near future, as there is little training available for clinical oncologists to develop expertise in genetic counselling and no new posts opening up in clinical genetics to meet this need.

The report also recommended that moderate-risk cases should usually be managed in the cancer units and primarily high-risk cases should be referred to the specialist genetics service. The proportion of population-risk cases attending the regional cancer genetics service ranged from 0 to 58% implying that the model has not yet been fully adopted. The extent to which population-level and moderate-risk individuals are dealt with in cancer units and primary care must vary between regions. However, we cannot be sure that individuals encountering these services are being managed optimally, given that most of these services are not, as yet, under the guidance of the specialist cancer genetics service, as envisaged by the Harper Report.

Evidence from The Netherlands suggests that cancer genetics practised in primary care may be less than optimal (de Bock et al, 2001), however, research-based initiatives are underway to educate primary care staff and facilitate liaison between the genetics services, cancer units and primary care and to reduce unnecessary referrals. These include the use of computerized risk

© 2001 Cancer Research Campaign 

assessment (Emery et al, 1999, 2000) or questionnaire-based family history assessment (Leggatt et al, 1999) by primary care teams. In several genetics services there are initiatives to train cancer unit nurses in genetic risk assessment and counselling. This will be strengthened with the proposed introduction of Macmillan funded posts into cancer genetic counselling.

Scotland appears to be closer to meeting the structure of its planned service model. There are few family history clinics outside of the regional genetics service. Consequently these services have more attendees than their English counterparts and proportionately more population-level and moderate-risk individuals. Of course, around the UK achievement of model guidelines may have improved since the survey was conducted.

### Appropriateness of service guidelines

The Scottish Office model emphasizes the importance of a central referral system for all potential cases of familial cancer and access to genetic counsellors for individuals at lower levels of risk. This is a more comprehensive but potentially more costly approach than that proposed by the Harper Report where population-level risk individuals are dealt with mainly in primary care and those at moderately elevated risk in the cancer unit. A recent clinical trial (Brain et al, 2000) has shown that multi-disciplinary genetics teams were no more effective than breast surgeons at managing cases of familial breast cancer, when effectiveness was measured in terms of psychosocial outcomes. This would suggest that the Scottish model is unnecessarily reliant on the clinical genetics service although it may not be possible to generalize the results of Brain et al's study to other regions. Furthermore, Brain et al may have underestimated the extent to which inappropriate resource allocation occurs when clinical genetics is not utilized. Some centres in our study were re-evaluating individuals whose risk has previously been overestimated by nonspecialists. We found that, across all regions, the geneticists were recommending that screening be discontinued for 19% of population-level risk individuals (and be avoided for the rest of this risk group).

Regardless of which service model is followed, it is important that there is cohesion between the clinical genetics service, cancer units and primary care and, at the time of this study, this seems to be more evident in Scotland than in the rest of the UK.

### Conclusions

The discovery of genes that cause elevated risk of cancer has lead to a substantial increase in the number of individuals being identified as having a 'genetic disorder'. This has established a workload, which is beyond the capacity of the UK regional genetics services. In the short term at least, most of these individuals must be dealt with by other sectors of the health service but to ensure appropriate clinical management this should be under the guidance of specialist cancer geneticists. The lack of cohesion between clinical cancer genetics and other cancer services is more evident in England and Wales than in Scotland. Initiatives to disseminate referral and management guidelines to cancer units and primary care should be encouraged and adequately resources so that clinical genetics teams can focus on genetic testing and management of high-risk families.

### ACKNOWLEDGEMENTS

The authors wish to express their gratitude to the following lead clinicians and their teams for their participation in the study: Carol Chu, Trevor Cole, Jacqueline Cook, Rosemarie Davidson, Fiona Douglas, Diana Eccles, Rosalind Eeles, Ian Ellis, Gareth Evans, Jonathon Gray, Neva Haites, Shirley Hodgson, Anneke Lucassen, James Mackay, Kay McDermott, Patrick Morrison, Victoria Murday, Mary Porteous, Sandy Raeburn, Michael Steel, Richard Trembath and Peter Turnpenny.

Thanks are expressed to Kathryn Ayres for clerical support and to Wendy Watson, Service user representative, and Hilary Harris, General Practitioner, for their advice. The Cancer Genetics Group of the British Society for Human Genetics (formerly the UK Cancer Family Study Group) is acknowledged for its support in promoting and supporting the research.

The research was funded by the NHS Research and Development Directorate, Programme for Cancer.

### REFERENCES

Brain K et al (2000) Randomized trial of a specialist genetic assessment service for familial breast cancer. *J Natl Cancer Inst* **92**: 1345–1351

de Bock GH, van Asperen CJ, de Vries JM, Hageman CHA, Springer MP and Kievit J (2001) How women with a family history of breast cancer and their general practitioners act on genetic advice in general practice: prospective longitudinal study. *BMJ* **7277**: 26–27

Eeles RA and Murday VA (1996) The cancer family clinic. In: Eeles RA, Ponder BAJ, Easton DF and Horwich A (Eds.) *Genetic Predisposition to Cancer*. London: Chapman & Hall

Emery J, Walton R, Coulson A, Glasspool D, Ziebland S and Fox J (1999) Computer support for recording and interpreting family histories of breast and ovarian cancer in primary care (RAGs): qualitative evaluation with simulated patients. *BMJ* **319**: 32–36

Emery J, Walton R, Murphy M, Austoker J, Yudkin P, Chapman C, Coulson A, Glasspool D and Fox J (2000) Computer support for interpreting family histories of breast and ovarian cancer in primary care: comparative study with simulated cases. *BMJ* **321**: 28–32

Expert Advisory Group on Cancer (1995) *A policy framework for commissioning cancer services*. London, Department of Health and Welsh Office

Hodgson S, Milner B, Brown I, Bevilacqua G, Chang-Claude J et al (1999) Cancer genetics services in Europe. *Disease Markers* **15**: 3–13

Leggatt V, Mackay J and Yates JRW (1999) Evaluation of a questionnaire on cancer family history in identifying patients at increased genetic risk in general practice. *BMJ* **319**: 757–758

Morrison PJ and Nevin NC (1999) Cancer genetics Services in Northern Ireland. *Disease Markers* **15**: 37–40

Moynihan C (1998) Theories in health care research: Theories of Masculinity. *BMJ* **317**: 1072–1075

Murday V (1994) Genetic counselling in the cancer family clinic. *European Journal of Cancer* **30A**: 2012–2015

NHS Executive (2000) *The NHS Cancer Plan. London*: Department of Health

Peshkin BN and Lerman C (1999) Genetic counselling for hereditary breast cancer. *The Lancet* **353**: 2176–2177

Ponder BAJ (1994) Setting up and running a familial cancer clinic. *British Medical Bulletin* **50**: 732–745

Priority Areas Cancer Team (1998) *Cancer Genetics Services in Scotland*. Edinburgh: The Scottish Office, Department of Health

Sobol H, Bignon Y-J, Bonaiti C, Cuisenier J, Lasset C et al (1999) Four year analysis of cancer genetic clinics activity in France from 1994 to 1997: A survey on 801 patients. *Disease Markers* **15**: 15–29

Thompson JA et al (1995) Genetics services for familial cancer patients: A survey of National Cancer Institute cancer centres *J Natl Cancer Inst* **87**: 1446–1455

Warner E, Foulkes W, Goodwin P, Meschino W, Blondal J, Paterson C, Ozcelik H, Goss P, Allingham-Hawkins D, Hamel N, Di-Prospero L, Contiga V, Serruya C, Klein M, Moslehi R, Honeyford J, Liede A, Glendon G, Brunet JS and Narod S (1999) Prevalence and penetrance of BRCA1 and BRCA2 gene mutations in unselected Ashkenazi Jewish women with breast cancer. *J Natl Cancer Inst* **91**: 1241–1247

Working Group for the Chief Medical Officer (1998) *Genetics and cancer services. Report of a Working Group for the Chief Medical officer, Department of Health*. London: Department of Health

 *© 2001 Cancer Research Campaign*
